# Treatment of class II malocclusion with Invisalign®: A pilot study using digital model-integrated maxillofacial cone beam computed tomography

**DOI:** 10.1016/j.jds.2022.08.027

**Published:** 2022-09-15

**Authors:** Shih-Yin Lin, Min-Chih Hung, Li-Hsin Lu, Jui-Sheng Sun, Shih-Jaw Tsai, Jenny Zwei-Chieng Chang

**Affiliations:** a Department of Dentistry, MacKay Memorial Hospital, Taipei, Taiwan; b School of Dentistry, College of Medicine, National Taiwan University, Taipei, Taiwan; c Department of Orthopedic Surgery, College of Medicine, China Medical University, Taichung, Taiwan; d Department of Orthopedic Surgery, National Taiwan University Hospital, Taipei, Taiwan

**Keywords:** Invisalign®, Class II malocclusion, ClinCheck®, Cone beam computed tomography, Digital model

## Abstract

**Background/purpose:**

The treatment effects of Invisalign® are still obscure due to methodological limitations of previous studies. We introduced a method to comprehensively evaluate the dental and skeletal changes of Class II malocclusion treated non-extraction with Invisalign® and compare with the virtual simulation of ClinCheck® using digital models integrated into maxillofacial cone-beam computed tomography (CBCT).

**Materials and methods:**

The pretreatment (T1) and posttreatment (T2) scanned digital images of actual dentitions were integrated into maxillofacial CBCT images. To evaluate three-dimensional movement of maxillary teeth and change of mandible position, T1 and T2 digital model-integrated maxillofacial CBCT images were superimposed using voxel-based registrations of stable cranial base structures. To evaluate movement of mandibular teeth, model-integrated mandibular CBCT superimposition was registered on mandibular basal bone. To compare achieved and predicted tooth movements, the actual dental images and the virtual digital models created by ClinCheck® were registered on the T1 dentitions.

**Results:**

For simulated upper first molar (U6) distalization of more than 1 mm, treatment accuracy ranged from 31.1% to 40.1%, which was significantly less than virtual planning and previous reports. In unilateral Class II subjects, the amount of U6 distalization on the Class II side was not significantly different from contralateral side, indicating efficacy of sequential distalization was questionable. Those with favorable overjet correction showed evidence of condylar distraction.

**Conclusion:**

Digital model-integrated CBCT superimpositions reflected the actual treatment changes in comparison with the virtual simulation, and showed that ideal occlusion was not achieved in mild to moderate Class II adult patients treated non-extraction with Invisalign®.

## Introduction

Since its development in 1997, Invisalign® has gained popularity because of improved esthetics and easier oral hygiene than conventional fixed orthodontic appliances.^1^,^2^ The combination of Invisalign® and Class II elastics has been established as a treatment option for Class II malocclusion.^3^ The manufacturer claims that Invisalign® effectively achieves distalization by using the sequential distalization protocol in combination with composite attachments and Class II elastics. Meta-analysis has shown more effective molar distalization using skeletal anchorage; on the contrary, premolar mesial movement was observed when using conventional anchorage.^4^ Theoretically, during sequential distalization, Invisalign® does not provide more distalization force/anchorage than other conventional devices; and after sequential distalization, the distalized teeth are prone to move mesially again to close the space created during distalization. It is dubitable whether the efficacy of distalization using Invisalign® is as high as claimed.

Studies have assessed the efficacy of clear aligners to achieve the expected/predicted goals of tooth movement in different directions, the differences of posttreatment results from that of fixed orthodontic devices, or the success of treatment using the American Board of Orthodontics (ABO) objective grading system (OGS).^5^ It is generally concluded that Invisalign® may achieve the treatment goals of non-extraction treatment for mild to moderate malocclusion in adults and the distalization of upper molars are highly predictable.^6^^,^^7^ However, studies using OGS have shown that clear aligners scores were consistently lower than braces scores in occlusal contacts, occlusal relationships, and overjet.^8^ No significant Class II correction or overjet reduction was observed for Class II adult patients treated with elastics at the end of first set of active aligners and all posttreatment occlusions failed to meet ABO standards.^9^

If distalization of maxillary molars with Invisalign® is highly predictable, how come OGS results show no significant Class II correction and overjet reduction? At present, no standard method exists for determining the efficacy of invisible orthodontic tooth movement.^6^ Most studies have compared the efficacy of tooth movement using superimposition of digital models. The references/registration of the superimposition included the occlusal plane,^10^^,^^11^ the teeth with less movement,^12^^,^^13^ and best-fit surface-based registration with software.^14^ These references are not stationary and may change after treatment. Simon et al. reported 88.4% accuracy for a planned upper molar distalization of 2.7 mm.^12^ The authors used the ‘untreated’ teeth as reference for superimposition. In the real-world scenario, the distal movement of the molars is balanced against the mesial movement of the teeth anterior to the molars. In the figure that the authors showed how they superimposed the digital scans, the incisors on the same side of molar distalization were retracted/distalized as well. There is a high possibility that the study has overestimated the efficacy of molar distalization. By using lateral cephalometric superimposition, Ravera et al. reported that non-extraction treatment of Class II malocclusion with Invisalign® and Class II elastics resulted in 2.25 mm distalization of maxillary first molars (U6).^15^ However, in the figure that the authors demonstrated their cephalometric superimposition, the posttreatment tracing (including the oribital rim, pterygomandibular fissure, coronoid process, and condylar head) all appeared to be more distally and superiorly positioned than the pretreatment cephalometric tracing. There is a high possibility of superimposition error and the amount of molar distalization and intrusion in that study may be overestimated. Another limitation of the cephalometric method is that the predicted virtual tooth movement cannot be superimposed with a lateral cephalogram; therefore the efficacy of tooth movement in directions other than anteroposterior cannot be determined.

Considering the aforementioned limitations of previous studies and that the Class II treatment effects of Invisalign® are still obscure, we aimed to analyze the non-extraction treatment outcomes of Class II adult patients treated with Invisalign® and Class II elastics after completion of the treatment with the initial set of clear aligners by using digital models integrated into maxillofacial cone beam computed tomography (CBCT). We investigated the changes in the upper and lower dentitions and mandible position, and compared with the virtual tooth movement in ClinCheck®. We further grouped the patients into good and bad responders of overjet correction and identified the associated variables that differentiated the two groups. We also examined the correlations between these variables and the amount of overjet correction.

## Materials and methods

This retrospective study included patients who had started treatment from January 2017 to December 2018 and completed the first series of Invisalign® performed by a sole orthodontist with approximately 10 years of Invisalign® experiences. The ethical approval was obtained from the Research Ethic Committee of National Taiwan University Hospital (approval number: 202108032RIND). All the methods were performed in accordance with the relevant guidelines and regulations. All clinical records used in this study were obtained from archives of the patients, from which informed consent forms were signed before any intervention, highlighting the possible use of materials from their records for research purposes.

The inclusion criteria were as follows:^1^ bilateral/unilateral Class II molar relationship as defined by the ABO,^2^ aged 18 years and older,^3^ non-extraction (except for the third molars),^4^ upper molar distalization with Class II elastics planned in ClinCheck®, and^5^ complete records of intraoral digital scans and maxillofacial CBCT images before (T1) and after (T2) treatment (the end of the first set of active aligners). The exclusion criteria were as follows:^1^ signs/symptoms of temporomandibular joint disorder,^2^ periodontal problems,^3^ congenital missing or impacted teeth (except for the third molar), and^4^ medication that could affect tooth movement.

**Figure 1:**
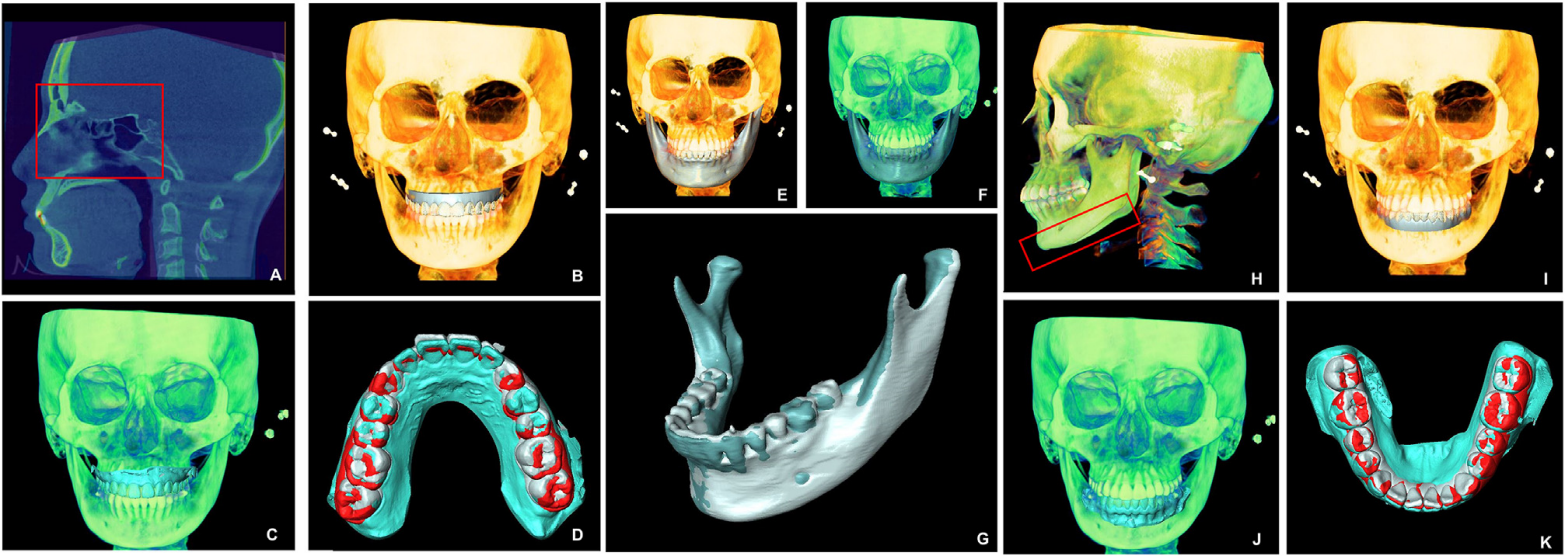
**Superimposition of the digital model-integrated maxillofacial cone beam computed tomography (CBCT) images and the dentitions. (A)** Superimposition of the pretreatment (T1) and posttreatment (T2) maxillofacial CBCT images by registration on the stable skeletal structures including the anterior cranial base, the frontal bone, and the periorbital bone. The red frame is the reference region of the superimposition. **(B)** Incorporation of the scanned digital image of the actual T1 maxillary dentition into the maxillofacial CBCT image at T1. **(C)** Incorporation of the scanned digital image of the actual T2 maxillary dentition into the maxillofacial CBCT image at T2. **(D)** Superimposition of T1 (gray), actual T2 (blue), and the virtual T2 (red) maxillary dentition from ClinCheck® by registration on the actual and virtual T1 maxillary dental arches while maintaining the voxel-based registrations of the anterior cranial base structures in (A). **(E)** Segmentation of the T1 mandible. **(F)** Segmentation of the T2 mandible. **(G)** Superimposition of the T1 and T2 mandibles while maintaining the voxel-based registrations of the anterior cranial base structures in (A). **(H)** Superimposition of the T1 and T2 CBCT images by registration on mandibular basal bone including the symphysis and the mandibular body. The red frame is the reference region of the superimposition. **(I)** Incorporation of the scanned digital image of the actual T1 mandibular dentition into the CBCT image at T1. **(J)** Incorporation of the scanned digital image of the actual T2 mandibular dentition into the CBCT image at T2. (**K**) Superimposition of T1 (gray), actual T2 (blue), and the virtual T2 (red) mandibular dentition from ClinCheck® by registration on the actual and virtual T1 mandibular dental arches while maintaining the registrations of the mandibular basal bone structures in (**H**).

The CBCT datasets at T1 and T2 in DICOM format were evaluated with Amira ver. 2020.3 (Thermo Fisher Scientific, Dallas, TX, USA). The overall superimposition was performed with the voxel-based method using the anterior cranial base, the frontal bone, and the periorbital bone as the registration structures. The scanned digital images of the actual maxillary dentitions at T1 and T2 in StereoLithography (STL) format were incorporated into the CBCT images at T1 and T2, respectively, via surface-based registration. The virtual pretreatment and posttreatment maxillary digital models created by ClinCheck® were exported in STL format. The virtual pretreatment and actual T1 maxillary dentitions were registered on the dental arch since they had the same dentition, and the final superimposition of the T1, T2, and the virtual posttreatment models of the maxillary dentition could be obtained (Fig. 1A–D). To evaluate the change of mandible position, segmentation of the T1 and T2 mandibles was performed while maintaining the voxel-based registrations of the cranial base surface (Fig. 1E–G). To evaluate the movement of the mandibular teeth, mandibular superimposition was performed by registration on the mandibular basal bone. The actual T1 and T2 digital models of the mandibular dentitions were incorporated into the CBCT images at T1 and T2, respectively. The virtual pretreatment and actual T1 mandibular dentitions were registered on the dental arch to obtain the final superimposition of the T1, T2, and virtual posttreatment models of the lower dentition (Fig. 1H–K).

**Figure 2:**
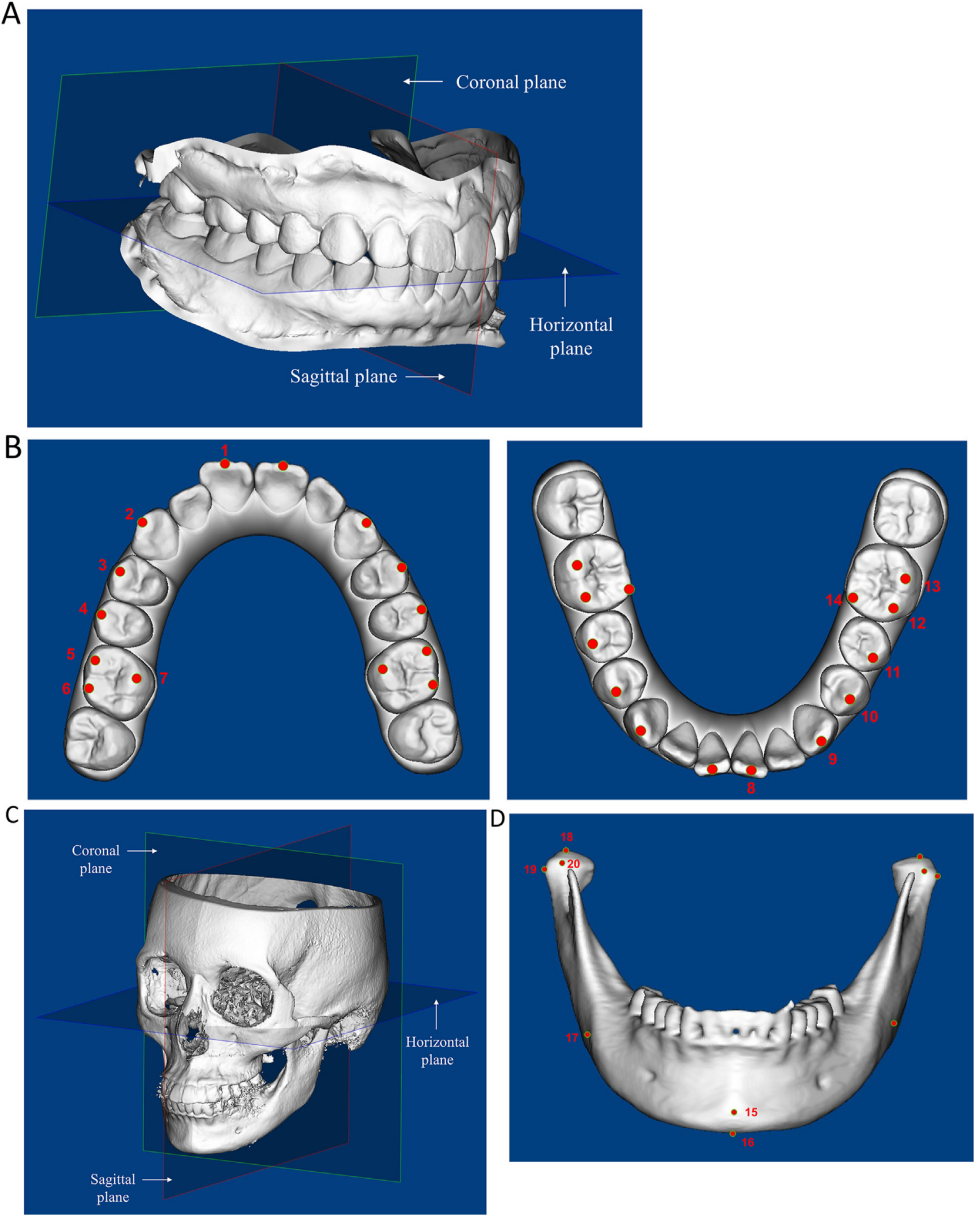
**Coordination systems and landmarks used to determine and describe tooth movement and change of mandible position. (A)** Three-dimensional coordinate system for tooth movement measurements. **(B)** The twenty-eight landmarks/points for measurement of tooth movement:^1^ midpoints of the incisal edge of the maxillary central incisors;^2^ the cusp tips of the maxillary canines;^3^ the buccal cusp tips of the maxillary first premolars;^4^ the buccal cusp tips of the maxillary second premolars;^5^ the mesiobuccal cusp tips of the maxillary first molars;^6^ the distobuccal cusp tips of the maxillary first molars;^7^ the mesiopalatal cusp tips of the maxillary first molars;^8^ midpoints of the incisal edge of the mandibular central incisors;^9^ the cusp tips of the mandibular canines;^10^ the buccal cusp tips of the mandibular first and^11^ second premolars; and the^12^ mesiobuccal,^13^ buccal, and^14^ mesiolingual cusps of the mandibular first molars. **(C)** Three-dimensional coordinate system for measurement of change in mandible position. **(D)** The ten anatomic landmarks of the mandible:^15^ pogonion;^16^ menton;^17^ gonion; and the^18^ uppermost,^19^ most lateral, and^20^ foremost points of the bilateral condylar heads.

After superimposition, the STL format of the superimposed images of T1, T2, and virtual post-treatment models were imported into Dolphin imaging premium ver. 11.95 (Dolphin Imaging & Management Solutions, Chatsworth, CA, USA) and a coordinate system was generated for tooth movement measurements (Fig. 2A). The horizontal plane was defined as the midpoint of the incisal edge of the upper right central incisor (U1) and the mesiobuccal (MB) cusps of the bilateral U6. The plane perpendicular to the horizontal plane and passing through the midpalatal suture was defined as the sagittal plane. The plane perpendicular to both horizontal and sagittal planes was obtained as the coronal plane. Twenty-eight landmarks of the upper and lower dentitions were identified (Fig. 2B). A second coordinate system was established for the measurements of mandible position (Fig. 2C). The Frankfurt horizontal plane was defined as the horizontal plane. The plane passing through the nasion and the basion and perpendicular to the horizontal plane was defined as the sagittal plane. The coronal plane was perpendicular to the two aforementioned planes. Ten anatomic landmarks of the mandible were identified (Fig. 2D). The coordinate values of the aforementioned landmarks were then output for subsequent data calculation.

The changes in the coordinate systems of the landmarks on the upper and lower dentitions indicated the three-dimensional movement of corresponding teeth. The difference between the virtual posttreatment model simulated by ClinCheck® and T1 was defined as the predicted tooth movement. The difference between T2 and T1 was defined as the achieved tooth movement. The percentage of treatment accuracy was defined as the (amount achieved)/(amount predicted)x100%. The changes of the coordinates of the mandible from T1 to T2 represented changes of the mandibular position during treatment. Additional measurements included the overjet, overbite, curve of Spee, and cephalometric variables obtained from CBCT-derived lateral cephalograms such as ANB angle, mandibular plane angle, U1-SN angle, and L1-MP angle.

The same observer identified all thirty-eight landmarks again two weeks later and comparisons were analyzed with the intraclass correlation coefficients (ICCs), which were excellent for most landmarks (ranging from 0.921 to 1.000) and good for the x-value of the MB cusp of the right U6 (0.881), the z-value of the DB cusp of the left U6 (0.870), and the x-value of the buccal cusp of the right L6 (0.804). The ICCs were exceptionally excellent for the uppermost and foremost landmarks of the condylar heads (0.996–1.000). Data were analyzed using SPSS 25.0 (IBM Corp., Armonk, NY, USA). The differences between the predicted and achieved amounts of tooth movement and the changes of cephalometric values, overjet, overbite, and the coordinates of the mandibular landmarks from T1 to T2 were evaluated using Wilcoxon signed rank tests. Those who had achieved normal overjet (the horizontal distance from the most labial side of the lower central incisor to the corresponding upper central incisor was between 1 and 3 mm) were defined as good responders, otherwise the bad responders.^16^ The Mann–Whitney U test was used to compare the good- and bad-responding groups. The relationships between overjet correction and the aforestated variables were accessed using the Pearson's correlation coefficient test. The significance level was set at *P*-value < 0.05.

## Results

**Figure 3:**
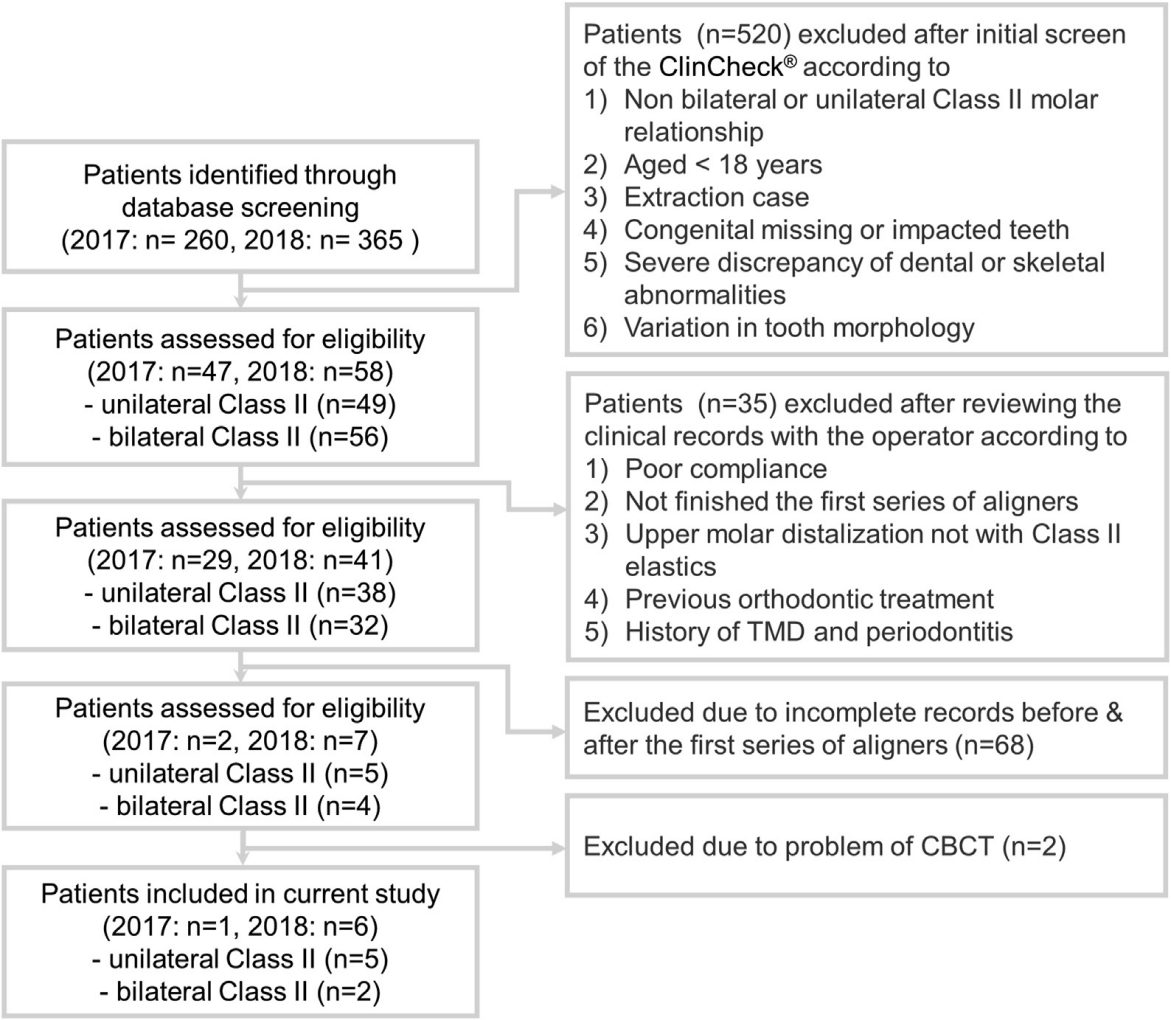
The sampling flow chart.

**Figure 4:**
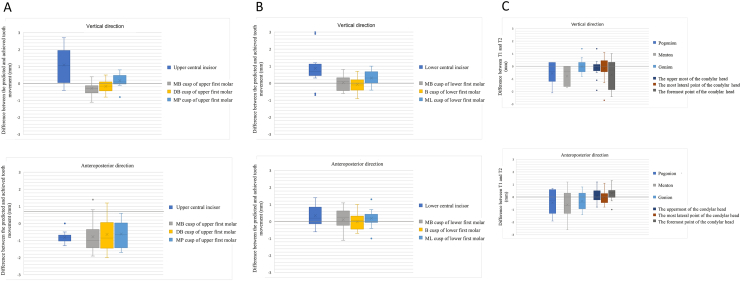
Distribution of the difference between the predicted and achieved tooth movements of (A) the maxillary dentition and (B) the mandibular dentition and the (C) changes in mandible position. The "x" mark represents the mean value. For **(A)** and **(B)**, the closer to 0-line, the higher the treatment accuracy. Above 0-line (+): more dental extrusion/retraction/distalization than predicted. Below 0-line (−): more dental intrusion/proclination/mesialization than predicted. For **(C)**, mandible upward/forward movement: +; downward/backward: -. Abbreviation: MB, mesiobuccal; DB, distobuccal; MP, mesiopalatal; B, buccal; ML, mesiolingual; T1, pretreatment; T2, posttreatment.

Only seven patients met the inclusion criteria (Fig. 3 and Table 1). After treatment, U1-SN angle significantly decreased and L1-MP angle significantly increased. The differences of achieved upper tooth movement from prediction and the percentage of treatment accuracy are shown in Table 2A and Fig. 4A. The treatment accuracies for maxillary intercanine, inter-1st-premolar, inter-2nd-premolar, and inter-1st-molar widths were 93.4%, 86.0%, 91.2%, 85.2%, respectively. The predicted upper central incisor movement was to intrude 0.35 ± 1.36 mm. However, it extruded 0.75 ± 1.51 mm. The actual amount of retraction/distalization was significantly less than planned. The accuracies of retraction of U1 and distalization of the MB cusp, DB cusp, and MP cusp of U6 were 50%, 46.5%, 56.5%, and 37.2%, respectively.

**Table 1:** General data and lateral cephalometric analysis at T1 and T2.

Age at T1 (years)	26.64 ± 3.02 (range 23.1–31.5)		
T2-T1 (months)	13.78 ± 8 (range 7.7–29.3)		
	T1	T2	*P*-value

ANB (°)	4.76 ± 1.92	5.13 ± 1.56	0.075
SN-MP (SN-GoGn) (°)	35.09 ± 4.43	35.6 ± 4.33	0.089
U1-SN (°)	109.06 ± 3.2	104.03 ± 3.65	0.018∗
L1-MP (°)	95.83 ± 3.6	102.4 ± 3.82	0.018∗
Overjet (mm)	4.53 ± 1.52	2.81 ± 1.1	0.063
Overbite (mm)	3.44 ± 2.14	2.31 ± 0.91	0.176

∗ Statistically significant difference between T1 and T2: *P* < 0.05, Number = 7

Abbreviation: T1, pretreatment; T2, after initial set/series of clear aligners; ANB, the angle formed by the anatomic landmarks nasion-A line and nasion-B line; SN-MP, the angle between the sella-nasion line and the mandibular plane (Gonion-Gnathion); U1-SN, the angulation of U1 axis to sella-nasion line; L1-MP, the angulation of lower central incisor to mandibular plane.

**Table 2A:** The difference between the predicted and achieved tooth movement of upper dentition and lower dentition.

Upper dentition						
		Predicted (mm)	Achieved (mm)	Difference (mm)	Accuracy	*P*-value
Horizontal direction (N = 7)	Upper intercanine width	1.94 ± 2.21	1.81 ± 2.02	−0.13 ± 0.59	93.4%	0.499
	Upper inter-first-premolar width	3.27 ± 2.42	2.81 ± 2.26	−0.46 ± 0.74	86.0%	0.128
	Upper inter-second-premolar width	1.98 ± 1.91	1.81 ± 1.7	−0.17 ± 0.73	91.2%	0.612
	Upper inter-first-molar width	2.1 ± 1.63	1.79 ± 1.29	−0.31 ± 0.97	85.2%	0.499
Vertical direction (N = 14)	Upper central incisor	−0.35 ± 1.36	0.75 ± 1.51	1.1 ± 1.03	−214.3%	0.006∗
	MB cusp of upper first molar	−0.07 ± 0.37	−0.36 ± 0.66	−0.29 ± 0.41	500.0%	0.035∗
	DB cusp of upper first molar	−0.2 ± 0.41	−0.36 ± 0.62	−0.16 ± 0.34	182.1%	0.106
	MP cusp of upper first molar	−0.16 ± 0.42	0.01 ± 0.68	0.18 ± 0.42	−8.7%	0.107
AP Direction (N = 14)	Upper central incisor	1.61 ± 0.8	0.81 ± 0.89	−0.81 ± 0.32	50.0%	0.001∗
	MB cusp of upper first molar	1.44 ± 0.91	0.67 ± 0.5	−0.77 ± 0.94	46.5%	0.020∗
	DB cusp of upper first molar	1.48 ± 0.78	0.84 ± 0.6	−0.64 ± 0.99	56.5%	0.034∗
	MP cusp of upper first molar	0.98 ± 0.85	0.36 ± 0.4	−0.61 ± 0.76	37.2%	0.017∗

Lower dentition						

		Predicted (mm)	Achieved (mm)	Difference (mm)	Accuracy	*P*-value

Horizontal direction (N = 7)	Lower intercanine width	1.96 ± 1.53	1.61 ± 1.38	−0.36 ± 0.54	81.8%	0.176
	Lower inter-first-premolar width	2.99 ± 2.25	2.5 ± 2.12	−0.49 ± 0.56	83.8%	0.043∗
	Lower inter-second-premolar width	1.3 ± 3.77	1.41 ± 3.12	0.11 ± 1.17	108.4%	0.866
	Lower inter-first-molar width	1.75 ± 1.86	1.8 ± 1.61	0.04 ± 0.68	102.4%	0.612
Vertical direction (N = 14)	Lower central incisor	−1.58 ± 1.26	−0.7 ± 0.83	0.88 ± 1.04	44.3%	0.008∗
	MB cusp of lower first molar	0.04 ± 0.38	0.07 ± 0.45	0.04 ± 0.43	200.0%	0.801
	Buccal cusp of lower first molar	−0.01 ± 0.27	−0.09 ± 0.36	−0.08 ± 0.41	650.0%	0.461
	ML cusp of lower first molar	0.13 ± 0.43	0.44 ± 0.38	0.31 ± 0.43	338.9%	0.027∗
AP Direction (N = 14)	Lower central incisor	−1.56 ± 1.12	−1.21 ± 0.86	0.35 ± 0.63	77.6%	0.071
	MB cusp of lower first molar	0.12 ± 0.97	0.23 ± 1.23	0.11 ± 0.65	188.2%	0.362
	Buccal cusp of lower first molar	0.29 ± 0.83	0.28 ± 0.94	−0.01 ± 0.51	95.1%	0.752
	ML cusp of lower first molar	−0.14 ± 1.11	0.05 ± 1.29	0.19 ± 0.53	−36.8%	0.132

Arch expansion: +; arch constriction: -

Teeth extrusion: +; teeth intrusion: -

Incisor retraction/molar distalization: +; incisor proclination/molar mesialization: -

Abbreviation: MB, mesiobuccal; DB, distobuccal; MP, mesiopalatal; ML, mesiolingual; N, number; AP, anteroposterior.

∗ Statistically significant difference: *P* < 0.05.

The differences of achieved lower tooth movement from prediction and the percentage of treatment accuracy are shown in Table 2A and Fig. 4B. The treatment accuracies for mandibular intercanine, inter-1st-premolar, inter-2nd-premolar, and inter-1st-molar widths were above 80%. The ML cusp of L6 extruded significantly more than planned. The lower central incisor was expected to intrude 1.58 ± 1.26 mm, however, it only intruded 0.7 ± 0.83 mm with accuracy of 44.3%. The positional changes of the mandibular skeletal landmarks during treatment are shown in Table 2B and Fig. 4C. The menton had moved significantly downward by 0.79 ± 0.78 mm and the foremost point of the condylar head moved significantly forward by 0.3 ± 0.57 mm.

**Table 2B:** The positional changes of the mandibular skeletal landmarks during treatment.

	Vertical direction (mm)	*P*-value	AP direction (mm)	*P*-value
Pogonion (N = 7)	−0.56 ± 0.86	0.128	−0.46 ± 0.97	0.237
Menton (N = 7)	−0.79 ± 0.78	0.043∗	−0.6 ± 1.2	0.237
Gonion (N = 14)	−0.02 ± 0.59	0.598	−0.31 ± 0.68	0.109
The uppermost point of condylar head (N = 14)	−0.12 ± 0.75	0.624	0.09 ± 0.49	0.384
The most lateral point of condylar head (N = 14)	−0.18 ± 0.97	0.861	−0.04 ± 0.53	0.694
The foremost point of condylar head (N = 14)	−0.66 ± 1.22	0.124	0.3 ± 0.57	0.042∗

Mandible upward/forward movement: +; downward/backward.

∗ Statistically significant difference: *P* < 0.05.

Abbreviation: N, number; AP, anteroposterior.

**Figure 5:**
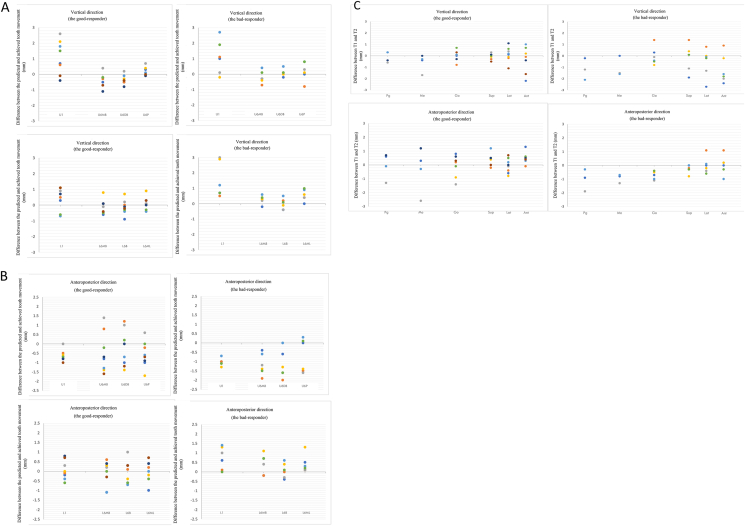
Comparison of the difference between the predicted and achieved tooth movements in the (A) vertical and (B) anteroposterior directions and the (C) change of mandible position in the good- and bad-responding groups. For **(A)** and **(B)**, the closer to 0-line, the higher the treatment accuracy. Above 0-line (+): more dental extrusion/retraction/distalization than predicted. Below 0-line (−): more dental intrusion/proclination/mesialization than predicted. For **(C)**, mandible upward/forward movement: +; downward/backward: -. Abbreviation: U1, the upper central incisor; U6MB, the mesiobuccal cusp of the upper first molar; U6DB, the distobuccal cusp of the upper first molar; U6P, the mesiopalatal cusp of the upper first molar; L1, the lower central incisor; L6MB, the mesiobuccal cusp of the lower first molar; L6B, the buccal cusp of the lower first molar; L6ML, the mesiolingual cusp of the lower first molar; Pg, pogonion; Me, menton; Go, gonion; Sup, the uppermost point of the condyle; Lat, the most lateral point of the condyle; Ant, the foremost point of the condyle; T1, pretreatment; T2, posttreatment.

To evaluate the factors that might have influenced the effectiveness of treatment, the patients were further divided into the good- or bad-responding groups for overjet correction. The two groups showed comparable cephalometric characteristics before treatment. Although statistically insignificant, the bad-responders tended to have more increase of the ANB and SN-MP (Table 3A). Both groups were planned to intrude L1. However, the good-responders exhibited 1.25 ± 0.54 mm of L1 intrusion (75.2% accuracy), whereas the bad-responders exhibited extrusion of 0.12 ± 0.78 mm (Table 3B and Fig. 5A). The good-responders showed better vertical control of the U6 DB cusp and the L6 ML cusp. The bad-responders had apparently less U6 distalization, U1 retraction, and L11 proclination than planned and more distalization of the L6; while the good-responders showed mesialization of the L6 ML cusp (Table 3B and Fig. 5B). With respect to the mandibular position, the good-responders showed a significant forward movement while the bad-responders showed a backward movement of the uppermost point of the condylar head (Table 3C and Fig. 5C).

**Table 3A:** Differences between the good- and bad-responding groups: general data and lateral cephalometric analysis.

	The good responders (N = 4)		The bad responders (N = 3)	
Age at T1	25.63 ± 1.56		28 ± 4.35	
T2-T1 (months)	9.82 ± 2.04		19.07 ± 10.6	

	**T1**	**T2**	**T1**	**T2**

ANB (°)	5.1 ± 1.81	5.35 ± 1.44	4.3 ± 2.35	4.83 ± 1.99
SN-MP (SN-GoGn) (°)	35.53 ± 6.22	35.65 ± 6.09	34.5 ± 0.26	35.53 ± 0.74
U1-SN (°)	110.4 ± 2.92	102.85 ± 4.14	107.27 ± 3.07	105.6 ± 2.8
L1-MP (°)	96.75 ± 2.64	103.83 ± 2.32	94.6 ± 4.95	100.5 ± 5.13
Overjet (mm)	4.7 ± 1.59	2.03 ± 0.6	4.3 ± 1.73	3.87 ± 0.46
Overbite (mm)	3.2 ± 2.96	1.95 ± 0.6	3.77 ± 0.57	2.8 ± 1.15

Abbreviation: T1, pretreatment; T2, after initial set/series of clear aligners; ANB, the angle formed by the anatomic landmarks nasion-A line and nasion-B line; SN-MP, the angle between the sella-nasion line and the mandibular plane (Gonion-Gnathion); U1-SN, the angulation of U1 axis to sella-nasion line; L1-MP, the angulation of lower central incisor to mandibular plane; N, number.

**Table 3B:** Differences of the tooth movement between the good- and bad-responding groups in the horizontal, vertical, and anteroposterior directions.

		The good responders (N = 4)					The bad responders (N = 3)				
		Predicted (mm)	Achieved (mm)	Difference (mm)	Accuracy	*P*-value	Predicted (mm)	Achieved (mm)	Difference (mm)	Accuracy	*P*-value
Horizontal direction	Upper intercanine width	3.27 ± 2.02	3.04 ± 1.63	−0.22 ± 0.41	93.1%	0.465	0.16 ± 0.54	0.16 ± 1.06	0 ± 0.87	101.3%	1.000
	Upper inter-first-premolar width	4.34 ± 2.84	3.75 ± 2.66	−0.6 ± 0.35	86.2%	0.068	1.83 ± 0.36	1.56 ± 0.81	−0.27 ± 1.17	85.3%	0.593
	Upper inter-second-premolar width	2.96 ± 1.92	2.6 ± 1.87	−0.36 ± 0.68	87.9%	0.465	0.67 ± 0.94	0.74 ± 0.67	0.07 ± 0.87	110.8%	0.593
	Upper inter-first-molar width	3.31 ± 0.83	2.58 ± 1.12	−0.73 ± 1.01	77.8%	0.273	0.48 ± 0.25	0.73 ± 0.45	0.25 ± 0.68	153.3%	0.593
	Lower intercanine width	1.37 ± 1.47	1.41 ± 1.41	0.05 ± 0.14	103.4%	0.715	0.16 ± 0.54	0.16 ± 1.06	0 ± 0.87	101.3%	0.109
	Lower inter-first-premolar width	2.75 ± 2.24	2.59 ± 2.18	−0.16 ± 0.25	94.0%	0.273	1.83 ± 0.36	1.56 ± 0.81	−0.27 ± 1.17	85.3%	0.109
	Lower inter-second-premolar width	3.56 ± 2.55	3.15 ± 2.91	−0.41 ± 0.73	88.4%	0.273	0.67 ± 0.94	0.74 ± 0.67	0.07 ± 0.87	110.8%	0.593
	Lower inter-first-molar width	2.99 ± 1.42	2.7 ± 1.61	−0.29 ± 0.63	90.2%	0.465	0.48 ± 0.25	0.73 ± 0.45	0.25 ± 0.68	153.3%	0.109

		The good responders (N = 8)					The bad responders (N = 6)				
		Predicted (mm)	Achieved (mm)	Difference (mm)	Accuracy	*P*-value	Predicted (mm)	Achieved (mm)	Difference (mm)	Accuracy	*P*-value

Vertical direction	Upper central incisor	0.13 ± 1.59	1.23 ± 1.78	1.1 ± 1.07	980.0%	0.036∗	−0.98 ± 0.65	0.12 ± 0.78	1.1 ± 1.09	−11.9%	0.075
	MB cusp of upper first molar	−0.21 ± 0.27	−0.56 ± 0.58	−0.35 ± 0.44	264.7%	0.067	0.12 ± 0.42	−0.08 ± 0.71	−0.2 ± 0.39	−71.4%	0.292
	DB cusp of upper first molar	−0.26 ± 0.3	−0.61 ± 0.52	−0.35 ± 0.3#	233.3%	0.025∗	−0.12 ± 0.55	−0.03 ± 0.63	0.08 ± 0.23#	28.6%	0.461
	MP cusp of upper first molar	−0.28 ± 0.27	−0.11 ± 0.43	0.16 ± 0.28	40.9%	0.168	−0.02 ± 0.55	0.18 ± 0.95	0.2 ± 0.6	−1100.0%	0.336
	Lower central incisor	−1.66 ± 1.06	−1.25 ± 0.54#	0.41 ± 0.71	75.2%	0.141	−0.98 ± 0.65	0.12 ± 0.78#	1.1 ± 1.09	−11.9%	0.027∗
	MB cusp of lower first molar	0.01 ± 0.39	−0.13 ± 0.38	−0.14 ± 0.47	−1000.0%	0.398	0.12 ± 0.42	−0.08 ± 0.71	−0.2 ± 0.39	−71.4%	0.058
	Buccal cusp of lower first molar	−0.06 ± 0.26	−0.19 ± 0.39	−0.13 ± 0.47	300.0%	0.352	−0.12 ± 0.55	−0.03 ± 0.63	0.08 ± 0.23	28.6%	1.000
	ML cusp of lower first molar	0.34 ± 0.16	0.46 ± 0.45	0.12 ± 0.4	137.0%	0.495	−0.02 ± 0.55	0.18 ± 0.95	0.2 ± 0.6	−1100.0%	0.042∗
		The good responders (N = 8)					The bad responders (N = 6)				
		Predicted (mm)	Achieved (mm)	Difference (mm)	Accuracy	*P*-value	Predicted (mm)	Achieved (mm)	Difference (mm)	Accuracy	*P*-value

AP direction	Upper central incisor	1.85 ± 0.81	1.21 ± 0.85#	−0.64 ± 0.3#	65.5%	0.018∗	1.3 ± 0.72	0.27 ± 0.67#	−1.03 ± 0.2#	20.5%	0.027∗
	MB cusp of upper first molar	1.31 ± 1.03	0.84 ± 0.53	−0.48 ± 1.08	63.8%	0.261	1.62 ± 0.78	0.45 ± 0.38	−1.17 ± 0.57	27.8%	0.028∗
	DB cusp of upper first molar	1.3 ± 0.8	1.06 ± 0.64	−0.24 ± 0.99	81.7%	0.497	1.72 ± 0.74	0.53 ± 0.42	−1.18 ± 0.74	31.1%	0.042∗
	MP cusp of upper first molar	0.86 ± 0.91	0.3 ± 0.45	−0.56 ± 0.7	34.8%	0.051	1.13 ± 0.84	0.45 ± 0.33	−0.68 ± 0.9	39.7%	0.225
	Lower central incisor	−1.64 ± 0.98	−1.58 ± 0.71	0.06 ± 0.5#	96.2%	0.735	−1.47 ± 1.38	−0.73 ± 0.86	0.73 ± 0.6#	50.0%	0.043∗
	MB cusp of lower first molar	−0.18 ± 0.61	−0.3 ± 0.67	−0.13 ± 0.66	171.4%	0.799	0.52 ± 1.27	0.93 ± 1.51	0.42 ± 0.53	180.6%	0.114
	Buccal cusp of lower first molar	0.05 ± 0.35	−0.02 ± 0.75	−0.08 ± 0.6	−50.0%	0.574	0.62 ± 1.18	0.68 ± 1.09	0.07 ± 0.39	110.8%	0.588
	ML cusp of lower first molar	−0.61 ± 0.59#	−0.6 ± 0.58#	0.01 ± 0.54	98.0%	0.798	0.5 ± 1.37#	0.92 ± 1.52#	0.42 ± 0.46	183.3%	0.027

Arch expansion: +; arch constriction: -

Teeth extrusion: +; teeth intrusion: -

Incisor retraction/molar distalization: +; incisor proclination/molar mesialization: -

Abbreviation: T1, pretreatment; T2, after initial set/series of clear aligners; MB, mesiobuccal; DB, distobuccal; MP, mesiopalatal; ML, mesiolingual; AP, anteroposterior; N, number.

∗ Statistically significant difference between T1 and T2: *P* < 0.05.

# Statistically significant difference between the good- and bad-responding groups, *P* < 0.05.

**Table 3C:** Differences of the mandibular position between the good- and bad-responding groups.

	The good responders					The bad responders			
	Vertical direction (mm)	*P*-value	AP direction (mm)	*P*-value		Vertical direction (mm)	*P*-value	AP direction (mm)	*P*-value
Pogonion (N = 4)	−0.1 ± 0.47	0.461	−0.03 ± 0.92	1.000	Pogonion (N = 3)	−1.17 ± 0.95	0.109	−1.03 ± 0.81	0.109
Menton (N = 4)	−0.6 ± 0.75	0.109	−0.35 ± 1.62	1.000	Menton (N = 3)	−1.03 ± 0.9	0.180	−0.93 ± 0.32	0.109
Gonion (N = 8)	−0.02 ± 0.45	0.865	−0.03 ± 0.76	0.779	Gonion (N = 6)	−0.02 ± 0.79	0.600	−0.68 ± 0.31	0.027∗
The uppermost point of condylar head (N = 8)	−0.09 ± 0.26	0.348	0.36 ± 0.44#	0.044∗	The uppermost point of condylar head (N = 6)	−0.17 ± 1.17	0.916	−0.27 ± 0.29#	0.066
The most lateral point of condylar head (N = 8)	0.13 ± 0.65	0.362	−0.06 ± 0.52	0.735	The most lateral point of condylar head (N = 6)	−0.58 ± 1.24	0.225	0 ± 0.6	0.686
The foremost point of condylar head (N = 8)	−0.27 ± 1.1	0.779	0.5 ± 0.39	0.017	The foremost point of condylar head (N = 6)	−1.18 ± 1.27	0.075	0.03 ± 0.69	0.892

Mandible upward/forward movement: +; downward/backward: -

Abbreviation: T1, pretreatment; T2, after initial set/series of clear aligners; AP, anteroposterior; N, number.

∗ Statistically significant difference between T1 and T2: *P* < 0.05.

# Statistically significant difference between the good- and bad-responding groups, *P* < 0.05.

Since unilateral Class II cases were also included in this study, bilateral Class II elastic wear and sequential distalization for the Class II side but not for the Class I side were planned. Based on the amount of U6 distalization planned in ClinCheck®, the data of the maxillary tooth movement in the anteroposterior direction was further divided into two groups: the intended-to-change and the intended-not-to-change groups (Table 3D). Although the intended-to-change group had planned a greater amount of U6 distalization, the actually achieved amount of U6 distalization was not significantly different between the two groups. The achieved amount of U6 distalization of the intended-to-change group was significantly less than originally planned, with treatment accuracy ranging from only 31.1%–40.1%.

**Table 3D:** Differences of the anteroposterior movement of teeth between the intended-to-change and the intended-not-to-change groups.

	Intended-to-change (greater than 1 mm distalization planned; N = 9)					Intended-not-to-change (less than 1 mm distalization planned; N = 5)				
	Predicted (mm)	Achieved (mm)	Difference (mm)	Accuracy	*P*-value	Predicted (mm)	Achieved (mm)	Difference (mm)	Accuracy	*P*-value
Upper central incisor	1.71 ± 0.89	0.79 ± 0.99	−0.92 ± 0.23	46.1%	0.008∗	1.44 ± 0.65	0.84 ± 0.78	−0.6 ± 0.38	58.3%	0.068
MB cusp of upper first molar	1.98 ± 0.62#	0.72 ± 0.48	−1.26 ± 0.5#	36.5%	0.008∗	0.48 ± 0.38#	0.58 ± 0.58	0.1 ± 0.94#	120.8%	0.686
DB cusp of upper first molar	1.97 ± 0.44#	0.79 ± 0.56	−1.18 ± 0.64#	40.1%	0.011∗	0.6 ± 0.24#	0.92 ± 0.73	0.32 ± 0.76#	153.3%	0.285
MP cusp of upper first molar	1.36 ± 0.82#	0.42 ± 0.31	−0.93 ± 0.68#	31.1%	0.017∗	0.3 ± 0.37#	0.26 ± 0.55	−0.04 ± 0.57#	86.7%	1.000

Incisor retraction/molar distalization: +; incisor proclination/molar mesialization: -

Abbreviation: T1, pretreatment; T2, after initial set/series of clear aligners; MB, mesiobuccal; DB, distobuccal; MP, mesiopalatal; N, number.

∗ Statistically significant difference between T1 and T2: *P* < 0.05.

# Statistically significant difference between the intended-to-change and the intended-not-to-change groups, *P* < 0.05.

To delineate which factors might have affected the correction of overjet, Pearson's correlation coefficient test was performed (Table 4). The variables that had reached statistical significance include overjet at T1, the predicted change of inter-U6-width, the actual change of inter-U6-width, the vertical change of U6 DB cusp, and the actual anteroposterior movement of L1.

**Table 4:** Pearson's correlation coefficients between overjet correction and other variables.

Variable	Pearson's correlation coefficient	*P*-value
T1 age	−0.008	0.987
T1 overjet	−0.761∗	0.047∗
T1 overbite	0.270	0.559
T1 ANB	−0.136	0.771
T1 SN-MP	−0.446	0.315
T1 U1-SN	−0.638	0.123
T1 L1-MP	0.313	0.494
T1 curve of Spee	−0.391	0.385
Predicted change of upper intercanine width	−0.552	0.199
Predicted change of upper inter-first-premolar width	−0.545	0.206
Predicted change of upper inter-second-premolar width	−0.598	0.156
Predicted change of upper inter-first-molar width	−0.815∗	0.025∗
Actual change of upper intercanine width	−0.694	0.084
Actual change of upper inter-first-premolar width	−0.659	0.108
Actual change of upper inter-second-premolar width	−0.667	0.102
Actual change of upper inter-first-molar width	−0.873∗	0.010∗
Predicted change of lower intercanine width	−0.012	0.980
Predicted change of lower inter-first-premolar width	0.008	0.987
Predicted change of lower inter-second-premolar width	−0.369	0.415
Predicted change of lower inter-first-molar width	−0.664	0.104
Actual change of lower intercanine width	−0.276	0.550
Actual change of lower inter-first-premolar width	−0.164	0.726
Actual change of lower inter-second-premolar width	−0.495	0.259
Actual change of lower inter-first-molar width	−0.691	0.085
Predicted vertical change of upper central incisor	−0.744	0.055
Actual vertical change of upper central incisor	−0.290	0.529
Actual vertical change of MB cusp of upper first molar	0.682	0.091
Actual vertical change of DB cusp of upper first molar	0.760∗	0.047∗
Actual vertical change of MP cusp of upper first molar	0.570	0.181
Actual anteroposterior change of upper central incisor	−0.497	0.256
Actual anteroposterior change of MB cusp of upper first molar	−0.227	0.624
Actual anteroposterior change of DB cusp of upper first molar	−0.258	0.577
Actual anteroposterior change of MP cusp of upper first molar	0.612	0.144
Actual vertical change of lower central incisor	0.559	0.192
Actual vertical change of MB cusp of lower first molar	0.301	0.512
Actual vertical change of buccal cusp of lower first molar	0.471	0.286
Actual vertical change of ML cusp of lower first molar	−0.304	0.508
Actual anteroposterior change of lower central incisor	0.847∗	0.016∗
Actual anteroposterior change of MB cusp of lower first molar	0.122	0.795
Actual anteroposterior change of buccal cusp of lower first molar	0.058	0.901
Actual anteroposterior change of ML cusp of lower first molar	0.249	0.590
Vertical difference in Pogonion	−0.550	0.201
Vertical difference in Menton	−0.290	0.528
Anteroposterior difference in Pogonion	−0.518	0.234
Anteroposterior difference in Menton	−0.496	0.257

Increased overjet and other cephalometric measurements: +; decreased: -

Upper and lower teeth expansion/extrusion/distalization: +; constriction/intrusion/mesialization: -

Mandible upward/forward movement: +; downward/backward: -

∗ Statistically significant difference: *P* < 0.05

Abbreviation: T1, pretreatment; ANB, the angle formed by the anatomic landmarks nasion-A line and nasion-B line; SN-MP, the angle between the sella-nasion line and the mandibular plane (Gonion-Gnathion); U1-SN, the angulation of U1 axis to sella-nasion line; L1-MP, the angulation of lower central incisor to mandibular plane; MB, mesiobuccal; DB, distobuccal; MP, mesiopalatal; ML, mesiolingual.

## Discussion

Previous Invisalign® studies have mostly adopted methods with certain errors or garnered results that could not be compared with ClinCheck® simulation. In this study, we are the first to analyze the treatment outcomes of Invisalign® by using digital models-integrated maxillofacial CBCT. Accurate registration of the two image-modalities is mandatory. The digital models may be integrated into the maxillofacial CBCT with minimal error using the surface-based registration method.^17^ Voxel-based method was used for skeletal superimposition because of the higher efficiency, elimination of segmentation errors in 3D surface models, and greater ease of assessing inner structures that allows the superimposed structures to be viewed in multiplanar slices.^18^^,^^19^

The average overjet decreased insignificantly from 4.53 ± 1.52 mm to 2.81 ± 1.1 mm after treatment (Table 1). The largest overjet in our patients was 7.1 mm and the molar relationship was half-cusp Class II. Therefore, the results of our study only represent the efficacy of the non-extraction treatment for mild to moderate Class II malocclusion. Invisalign® treatment typically required additional aligners for Class II malocclusion.^9^^,^^13^ All of the patients in this study were subsequently prescribed with additional aligners, indicating that completing the first series of aligners alone was insufficient to achieve the treatment goals. On the intended-to-change side, the treatment accuracy of U6 distalization ranged from only 31.1%–40.1% for the individual cusps, which was significantly less than originally planned. In comparison with the intended-not-to-change side, the actual amount of U6 distalization was not different between both sides. Since the intended-to-change side had received sequential distalization in ClinCheck® simulation, our results show that the efficacy of sequential distalization may be questionable. In our study, we superimposed on the stable bony structure and the results showed less molar distalization and accuracy compared with previous studies.

The mandible position may be affected by the vertical and horizontal vectors of the Class II elastics indirectly via extrusion of lower posterior teeth or directly via posturing forward (condylar distraction). In our study, 3D superimposition showed significant downward movement of menton by 0.79 ± 0.78 mm and insignificant backward movement by 0.6 ± 0.12 mm, whereas the foremost point of condyle had a significant forward movement of 0.3 ± 0.57 mm. In contrast with other studies, a tendency for the mandible to rotate backward and downward was observed, which might be caused by the significant extrusion of the L6 ML cusp. Since the mandible also postured forward a bit, the backward movement of the mandible became less significant. Favorable vertical control was reported by Ravera et al. and Caruso et al., whose patients all had completed treatment with additional aligners.^15^^,^^20^ However, as previously mentioned in the Introduction section, Ravera et al. seemed to have more distally and superiorly positioned their posttreatment tracing during cephalometric superimposition.^15^ While it seemed illogical that Caruso et al. showed a decrease of ANB angle but unchanged vertical skeletal measurements (such as SN-GoGn angle and N–Me) and a decrease of the SN-PP angle in their adult patients after treatment.^20^ Therefore, possible cephalometric superimpositional errors in these studies might have skewed the results.

Orthodontists often judge Class II treatment effectiveness based on the correction of overjet.^21^ In this study, the good-responders of overjet correction showed significantly more retraction of U1, intrusion of U6 DB cusp and L1, proclination of L1, and more anterior movement of the uppermost point of condylar head (condylar distraction). Pearson's correlation coefficient test showed that the larger the pretreatment overjet, the greater amount of predicted and achieved arch expansion at U6, the more intrusion of the U6 DB cusp, and the more proclinication of L1 would favor greater amount of overjet correction. Since the efficacy of molar distalization in this study was not satisfactory, its correlation with the amount of overjet correction was low. Both the good- and bad-responders showed approximately 1 mm more extrusion of U1 than planned, possibly due to effects of Class II elastics; while the L1 of bad-responders was also significantly more extruded than planned and than that of the good-responders. Consequently, anterior bite deepening/interference might have also caused mandible to rotate more backward and downward in the bad-responding group. Planning overcorrection of upper/lower incisor intrusion in ClinCheck® and using skeletal anchorage to facilitate anterior intrusion and total arch distalization should be considered in future when indicated.

The insufficient sample size was the greatest limitation of this study. All cases were treated by a single experienced orthodontist, who has held lectures for Align Technology and is the Black Diamond Invisalign® Provider and advisor of Invisalign® Academic Advisory Board. We believe that the results of this study objectively represent the standard results of non-extraction adult Class II treatment with Invisalign® and Class II elastics. Despite that, after screening all the 2017–2018 patients, only seven patients met the inclusion criteria. Due to the small sample size of this study, we considered this a pilot study and estimated the smallest sample size required for future studies. The main parameter of interest was the difference between the predicted and achieved distalization of the U6. Power analysis indicated that at least 21 samples (11 patients) were required to observe significant differences in all three cusps. To detect differences of at least one cusp greater than 1 mm, seven samples (4 patients) should be required. Despite the limitation, with digital model-integrated CBCT images, we are the first to comprehensively evaluated the dental and skeletal changes of the treatment by superimposing three-dimensionally on the stable bony structures and compare them with the results simulated by ClinCheck®. This method appears feasible for evaluation of other types of dental and skeletal malocclusions treated with Invisalign®.

## Declaration of competing interest

The authors declare no conflict of interest. Although Dr. S-J Tsai has held lectures and conferences for Align Technology in the past 10 years and is the Black Diamond Invisalign® Provider and advisor of Invisalign® Academic Advisory Board, this study was conducted without any support (financial or technical) from Align Technology. Dr. S-J Tsai was also not involved in the study design and analysis of the data.
